# Synthesis and *in-vitro* Evaluation of S-allyl Cysteine Ester - Caffeic Acid Amide Hybrids as Potential Anticancer Agents

**DOI:** 10.22037/ijpr.2019.15184.12921

**Published:** 2019

**Authors:** Wilson Castrillón, Angie Herrera-R, Laura Juliana Prieto, Laura Conesa-Milián, Miguel Carda, Tonny Naranjo, Maria Elena Maldonado, Wilson Cardona-G

**Affiliations:** a *Química de Plantas Colombianas, Institute of Chemistry, Faculty of Exact and Natural Sciences, University of Antioquia (UdeA), Calle 70 No. 52–21, A.A 1226, Medellín, Colombia. *; b *Grupo Impacto de los Componentes Alimentarios en la Salud, School of Nutrition and Dietetics, University of Antioquia (UdeA), A.A. 1226 Medellín. *; c *Department of Inorganic and Organic Chemistry, University Jaume I, E-12071 Castellón, Spain. *; d *Grupo de Micología Médica y Experimental, Corporación para Investigaciones Biológicas, Medellín, Colombia.*; e *School of Health Sciences, Universidad Pontificia Bolivariana, Medellín, Colombia.*; f *W. C. and A. H-R. contributed equally to this work.*

**Keywords:** S-allyl cysteine, Caffeic acid, Hybrid, Cell death, Colorectal cancer

## Abstract

We have synthesized a series of S-allyl cysteine ester-caffeic acid amide hybrids and evaluated them in order to determine their possible anticancer activity and selectivity in colorectal cancer, which is still one of the leading causes of morbidity and mortality worldwide. All compounds were tested against SW480 human colon adenocarcinoma cells and the non-malignant CHO-K1 cell line. Among the tested compounds, hybrids **6e**, **9a**, **9b**, **9c**, and **9e** exhibited the highest effect on viability (IC_50 SW480-48h_= 0.18, 0.12, 0.12, 0.11, and 0.12 mM, respectively) and selectivity (SI = 10.3, 1.5, >83.33, >90.91 and >83.33, respectively) in a time- and concentration-dependent manner. Besides, our results were even better as regards lead compounds (S-allyl cysteine and caffeic acid) and the standard drug (5-FU). Additionally, these five compounds induced mitochondrial depolarization that could be related with an apoptotic process. Moreover, hybrids **6e**, **9a**, and **9e** induced cell cycle arrest in G_2_/M phase, and compound **9c** in S- phase, which suggests that these hybrid compounds could have also a cytostatic effect in SW480 cell line. The SAR analysis showed that hydroxyl groups increased the activity. Besides, there was not a clear relationship between the antitumor properties and the length of the alkyl chain. Since hybrid compounds were much more selective than the conventional drug (5-FU), this makes them promising candidates for further studies against colorectal cancer.

## Introduction

Although colorectal cancer (CRC) can be prevented by healthy lifestyle habits this disease is still one of the leading causes of morbidity and mortality worldwide (1). In the most recent report of GLOBOCAN, the estimated burden of CRC until 2018 has exhibited an important increase through the years, being now the second most lethal cancer, preceded only by lung cancer. In addition, CRC is still the third most commonly diagnosed malignancy worldwide (2,3). Current chemotherapy for CRC involves multi-drug treatments such as FOLFIRI (folic acid/5-FU/irinotecan) and FOLFOX (5-FU/leucovorin/oxaliplatin) which are composed of 5-fluorouracil as the backbone of treatment. Although these therapies are effective, there are many undesirable gastrointestinal and neurological side effects associated with these treatments, which many times result in dose limitations or cessation of the anti-cancer therapy (4,5). Given the toxicity of the current chemotherapy, it is necessary to discover new, more potent and selective agents for treating this disease. According to this, the studies dealing with hybrid compounds have been reported as a promising strategy (6), since these molecules may display a dual activity (7,8).

S-allyl cysteine (SAC), a naturally occurring water-soluble constituent of garlic, has exhibited antioxidant properties both* in-vitro* (9,10) and *in-vivo* (11-13). Furthermore, SAC displayed antiproliferative effects on neuroblastoma (14) and melanoma (15). Additionally, in prostate carcinoma cells this compound induced cell cycle arrest at the G0/G1 phases and cell apoptosis with decreased in Bcl-2 expression and increased expression of Bax and caspase 8 (16,17). Although this compound did not show growth-inhibitory effects in HCT15 colon cancer cells or in lung and skin carcinoma cell lines (18), its administration prior to 1,2-dimethylhydrazine (DMH) injection significantly inhibited colonic nuclear damage in female in a dose–dependent manner. These data show that the allyl group coupled to a single sulfur atom might play an important structural role in the inhibition of chemical toxicity and carcinogenesis induced with DMH in the colon (19). 

**Figure 1:**
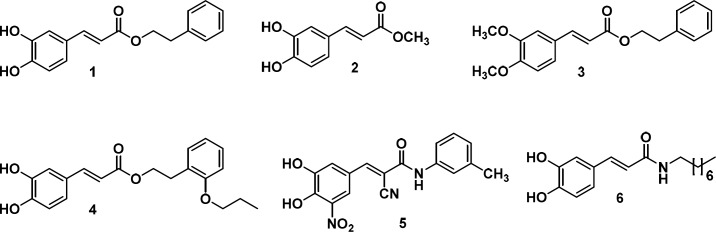
Caffeic acid derivatives endowed with antitumoral activity

On the other hand, caffeic acid and its derivatives display a broad spectrum of biological properties, among them, antioxidant (20,21), cytotoxic (22), pro-apoptotic, anti-inflammatory, and anti-angiogenic activity (23). Caffeic acid phenylethyl caffeate (CAPE, **1**), Methyl caffeate **2** and Phenylethyl dimethyl caffeate **3** (Figure1) significantly inhibited cell growth and syntheses of RNA, DNA, and protein of HT-29 colon adenocarcinoma cells. Moreover, when CAPE was tested in CT26 colon adenocarcinoma cells, it exhibited a decrease in cell viability in a dose–dependent manner, without displaying significant influence on the growth of human umbilical vein epithelial cells (24). Additionally, high anti-proliferative activity was observed when 2´-propoxyl CAPE derivative **4** was tested against Hela and DU-145 human cancer cell lines (25). Furthermore, treatment with CAPE reduced the formation of aberrant crypt foci (ACF) and tumor in the rat colon (26). In addition, caffeic acid was found to be the least effective growth inhibitor of HT-29 cells when compared to its ester analogs, highligting the potency of the derivatives (27). In this sense, cyanoamide derivative **5** showed *in-vitro* inhibitory activities against human gastric carcinoma cell line BGC-823, human nasopharyngeal carcinoma cell line KB, and human hepatoma cell line BEL-7402 with IC_50_ values of 5.6 µg/mL, 13.1 µg/mL, and 12.5 µg/mL, respectively (28). Finally, caffeic acid amide **6** and CAPE exhibited proapoptotic activity through activation of caspase-8 (29). 

**Figure 2:**
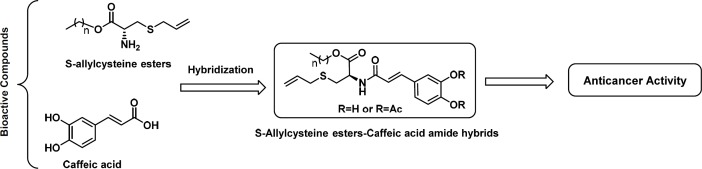
Design of S-allyl cysteine ester-caffeic acid amide hybrids as anti-cancer agents

There is an emerging strategy in medicinal chemistry and drug discovery research based on obtaining hybrid molecules that combine two or more structural fragments of the drugs that have a relevant pharmacological action (30,31). These hybrids may display dual activity but do not necessarily act on the same biological target (6,7). We have synthesized several S-allyl cysteine ester–caffeic acid amide hybrids (Figure 2) and their effect on proliferation, mitochondrial membrane permeability, and cell cycle distibution was determined in order to identify posible therapeutic approaches for the treatment of colorectal cancer.

## Experimental section

*Chemical synthesis*

*General remarks*

Microwave reactions were carried out in a CEM Discover microwave reactor in sealed vessels (monowave, maximum power 300 W, temperature control by IR sensor, and fixed temperature). ^1^H and ^13^C NMR spectra were recorded on a Varian instrument operating at 300, 600 and 75, 125 MHz, respectively. The signals of the deuterated solvent (CDCl_3 _or DMSO-D_6_) were used as reference. Chemical shifts (δ) are expressed in ppm with the solvent peak as reference and TMS as an internal standard; coupling constants (J) are given in Hertz (Hz). Carbon atom types (C, CH, CH_2_, CH_3_) were determined by using the DEPT pulse sequence. The signals were assigned using two-dimensional heteronuclear correlations (COSY, HSQC and HMBC). High resolution mass spectra were recorded using electrospray ionization mass spectrometry (ESI-MS). A QTOF Premier instrument with an orthogonal Z-spray-electrospray interface (Waters, Manchester, UK) was used operating in the W-mode. The drying and cone gas was nitrogen set to flow rates of 300 and 30 L/h, respectively. Methanol sample solutions (ca. 1 × 10^−5^ M) were directly introduced into the ESI spectrometer at a flow rate of 10 µL/min. A capillary voltage of 3.5 kV was used in the positive scan mode, and the cone voltage set to *U*c = 10 V. For accurate mass measurements, a 2 mg/L standard solution of leucine enkephalin was introduced via the lock spray needle at a cone voltage set to 85 V and a flow rate of 30 μL/min. IR spectra were recorded on a Spectrum RX I FT-IR system (Perkin-Elmer, Waltham, MA, USA) in KBr disks. Optical rotations were measured (Na-D line) at 25°C using a cell with 1dm path length on a Polartronic (Jasco model p-2000) polarimeter. Silica gel 60 (0.063–0.200 mesh, Merck, Whitehouse Station, NJ, USA) was used for column chromatography, and precoated silica gel plates (Merck 60 F254 0.2 mm) were used for thin layer chromatography (TLC). Monitoring of the reaction progress and product purification was carried out by TLC. 

*Procedure for the synthesis of S-Allylcysteine (* ***2*** *):* S-Cysteine hydrochloride (1 g, 6.34 mmol) was added to allyl bromide (1.15 g, 823 µL, 9.51 mmol) in 2M NH_4_OH (20 mL). The resulting mixture was stirred at room temperature for 20h. Then, the reaction mixture was concentrated to precipitate the product as a white solid. The solid was filtered, washed with ethanol (3 × 10 mL) and dried under reduced pressure, affording 818 mg (80%) of compound **2**. This compound was used in the following step without further purification.

^1^H NMR (DMSO-D_6_, 300 MHz): δ 2.48 (NH_2_), 2.72 (1H, dd, *J* = 14.6, 8.0 Hz, S-C**H**_2_CHN), 2.87 (1H, dd, *J* = 14.6, 4.2 Hz, S-C**H**_2_CHN), 3.06 (2H, d, *J* = 7.3 Hz, S-C**H**_2_CH=CH_2_), 3.56 (1H, dd, *J* = 8.0, 4.2 Hz, -C**H**-N), 4.29 (-NH_2_), 4.98-5.13 (2H, m, S-CH_2_CH=C**H**_2_), 5.59-5.75 (2H, m, S-CH_2_C**H**=CH_2_); ^13^C NMR (CDCl_3_, 75 MHz): δ 31.33 (S-**C**H_2_CHN), 33.93 (S-**C**H_2_CH=CH_2_), 53.45 (**C**H-N), 118.48 (S-CH_2_CH=**C**H_2_), 133.77 (S-CH_2_**C**H=CH_2_), 171.33 (-C=O)

*Procedure for the synthesis of S-Allylcysteine methyl ester (* ***3a*** *):*

Thionyl chloride (442.6 mg, 3.72 mmol, 270 µL) was added over 5 min. to dry methanol (15 mL) cooled to -10 °C and the resulting solution was stored for a further 5 min. Then, S-allyl cysteine (500 mg, 3.1 mmol) was added and the mixture was stirred for 10 min. The resulting solution was stored at -10 °C for 2h, kept at room temperature for other 24 h, and then poured into ether (100 mL) and refrigerated for 2 h. The product (488 mg, 90%) separated as colorless needles, was removed by 

filtration.

M.p. 114-116 °C; [α]^25^ + 4.778 (C = 0,013, CHCl_3_); IR (KBr, cm^-1^): ν _max _ 3366 (N-H), 1736 (C=O), 1238 (C-O-C); ^1^H NMR (DMSO-D_6_, 300 MHz): δ 2.51 (NH_2_), 2.84 (1H, dd, *J* = 14.7, 5.0 Hz, S-C**H**_2_CHN), 2.93 (1H, dd, *J* = 14.7, 5.0 Hz, S-C**H**_2_CHN), 3.09 (2H, d, *J* = 7.3 Hz, S-C**H**_2_CH=CH_2_), 3.70 (3H, s, OCH_3_), 4.14 (1H, dd, *J* = 7.1, 5.0 Hz, -C**H**-N), 5.04-5.15 (2H, m, S-CH_2_CH=C**H**_2_), 5.59-5.77 (1H, m, S-CH_2_C**H**=CH_2_); ^13^C NMR (CDCl_3_, 75 MHz): δ 30.09 (S-**C**H_2_CHN), 34.20 (S-**C**H_2_CH=CH_2_), 52.09 (O**C**H_3_), 54.52 (**C**H-N), 118.86 (S-CH_2_CH=**C**H_2_), 133.37 (S-CH_2_**C**H=CH_2_), 168.96 (-C=O). *EIMS: m/z *176,0732 [M + H]^+^*, *Calcd for C_7_H_14_NO_2_S: 176.0354.

*General procedure for the synthesis of S-Allyl cysteine esters (* ***3b-3e*** *):*

Thionyl chloride (3eq) was added over 5 min. to dry ethyl, propyl, butyl, or pentyl alcohol (15 mL) cooled to -10 °C and the resulting solution was stored for a further 5 min. Then, S-allyl cysteine (500 mg, 3.1 mmol) was added and the resulting mixture was stored at -10 °C for 2 h and the kept at room temperature for a further 24 h. Then the excess of alcohol was removed by distillation. The residue was purified by column chromatography over silica gel eluting with dichloromethane-methanol (95:5 ratio) to obtain S-allyl cysteine ethyl ester, S-allyl cysteine propyl ester, S-allyl cysteine butyl ester and S-allyl cysteine pentyl ester in 60% (352 mg), 71% (447 mg), 621% (418 mg) and 85% (609 mg) yields, respectively. Monitoring the reaction progress and product purification was carried out by TLC.

*Ethyl S-prop-2-en-1-ylcysteinate (* ***3b*** *):* M.p. 121-123°C; [α]^25^ + 1.40 (C = 0,62, CHCl_3_); IR (KBr, cm^-1^): ν _max _ 3472 (N-H), 1748 (C=O), 1231 (C-O-C); ^1^H NMR (CDCl_3_, 600 MHz): δ 0.96 (3H, t, *J* = 7.2 Hz), 2.63 (NH_2_), 3.22-3.25 (2H, m, S-C**H**_2_CHN), 3.18-3.22 (2H, m, S-C**H**_2_CH=CH_2_), 3.29 (1H, dd, *J* = 7.5, 5.0 Hz, -C**H**-N), 4.29 (2H, q, *J* = 7.0 Hz, OCH_2_), 5.15 (1H, d, *J* = 10 Hz, S-CH_2_CH=C**H**_2_), 5.24 (1H, d, *J* = 18 Hz, S-CH_2_CH=C**H**_2_), 5.74-5.85 (1H, m, S-CH_2_C**H**=CH_2_); ^13^C NMR (CDCl_3_, 125 MHz): δ 14.05 (CH_3_), 30.48 (S-**C**H_2_CHN), 35.10 (S-**C**H_2_CH=CH_2_), 52.69 (**C**H-N), 62.94 (OCH_2_), 118.51 (S-CH_2_CH=**C**H_2_), 133.37 (S-CH_2_**C**H=CH_2_), 167.95 (-C=O). *EIMS: m/z *190,0879 [M + H]^+^*, *Calcd for C_8_H_16_NO_2_S: 190.0356.

*Propyl S-prop-2-en-1-ylcysteinate (* ***3c*** *):* M.p. 115-117 °C; [α]^25^ + 3.750 (C = 0,015, CHCl_3_); IR (KBr, cm^-1^): ν _max _ 3375 (N-H), 1736 (C=O), 1182 (C-O-C); ^1^H NMR (CDCl_3_, 300 MHz): δ 0.91 (3H, t, *J* = 7.5 Hz), 1.53-1.72 (2H, m), 1.98 (NH_2_), 2.83 (1H, dd, *J* = 13.5, 5.0 Hz, S-C**H**_2_CHN), 2.65 (1H, dd, *J* = 13.5, 5.0 Hz, S-C**H**_2_CHN), 3.11 (2H, d, *J* = 7.0 Hz, S-C**H**_2_CH=CH_2_), 3.58 (1H, dd, *J* = 7.4, 5.0 Hz, -C**H**-N), 4.05 (2H, t, *J* = 6.7 Hz, OCH_2_), 5.07-5.16 (2H, m, S-CH_2_CH=C**H**_2_), 5.64-5.82 (1H, m, S-CH_2_C**H**=CH_2_); ^13^C NMR (CDCl_3_, 75 MHz): δ 10.41 (CH_3_), 21.97 (CH_2_), 35.14 (S-**C**H_2_CHN), 35.82 (S-**C**H_2_CH=CH_2_), 54.11 (**C**H-N), 66.82 (OCH_2_), 117.61 (S-CH_2_CH=**C**H_2_), 134.01 (S-CH_2_**C**H=CH_2_), 174.15 (-C=O). EIMS: m/z 204,1036 [M + H]^+^, Calcd for C_9_H_18_NO_2_S: 204.0603.

*Butyl S-prop-2-en-1-ylcysteinate (* ***3d*** *):* M.p. 101-103°C; [α]^25^ + 3.40 (C = 0,57, CHCl_3_); IR (KBr, cm^-1^): ν _max _ 3396 (N-H), 1750 (C=O), 1233 (C-O-C); ^1^H NMR (CDCl_3_, 600 MHz): δ 0.93 (3H, t, *J* = 7.5 Hz), 1.34-1.43 (2H, m), 1.62-1.70 (2H, m), 2.62 (NH_2_), 3.19 (2H, d, *J* = 6.8 Hz, S-C**H**_2_CH=CH_2_), 3.23 (1H, dd, *J* = 13.9, 7.0 Hz, S-C**H**_2_CHN), 3.29 (1H, dd, *J* = 13.9, 7.0 Hz, S-C**H**_2_CHN), 4.17-4.28 (1H, m, -C**H**-N), 4.38 (2H, t, *J* = 6.5 Hz, OCH_2_), 5.14 (1H, d, *J* = 10 Hz, S-CH_2_CH=C**H**_2_), 5.24 (1H, d, *J* = 17 Hz, S-CH_2_CH=C**H**_2_), 5.74-5.83 (1H, m, S-CH_2_C**H**=CH_2_); ^13^C NMR (CDCl_3_, 125 MHz): δ 13.66 (CH_3_), 19.02 (2CH_2_), 30.53 (S-**C**H_2_CHN), 35.11 (S-**C**H_2_CH=CH_2_), 52.67 (**C**H-N), 66.75 (OCH_2_), 118.49 (S-CH_2_CH=**C**H_2_), 133.38 (S-CH_2_**C**H=CH_2_), 168.08 (-C=O). EIMS: m/z 218,1206 [M + H]^+^, Calcd for C_10_H_20_NO_2_S: 218.0457.

*Pentyl S-prop-2-en-1-ylcysteinate (* ***3e*** *):* M.p. 98-100°C; [α]^25^ + 1.558 (C = 0,018, CHCl_3_); IR (KBr, cm^-1^): ν _max _ 3448 (N-H), 1747 (C=O), 1234 (C-O-C); ^1^H NMR (CDCl_3_, 600 MHz): δ 0.93 (3H, t, *J* = 7.0 Hz), 1.31-1.42 (4H, m), 1.60-1.74 (2H, m), 1.82 (NH_2_), 2.71 (1H, dd, *J* = 13.5, 5.3 Hz, S-C**H**_2_CHN), 2.90 (1H, dd, *J* = 13.5, 5.3 Hz, S-C**H**_2_CHN), 3.18 (2H, d, *J* = 7.17 Hz, S-C**H**_2_CH=CH_2_), 3.60-3.69 (1H, m, -C**H**-N), 4.16 (2H, t, *J* = 6.7 Hz,-OCH_2_), 5.11-5.20 (2H, m, S-CH_2_CH=C**H**_2_), 5.72-5.89 (1H, m, S-CH_2_C**H**=CH_2_); ^13^C NMR (CDCl_3_, 125 MHz): δ 13.70 (CH_3_), 22.03 (CH_2_), 27.78 (CH_2_), 28.01 (CH_2_), 34.83 (S-**C**H_2_CHN), 35.53 (S-**C**H_2_CH=CH_2_), 54.84 (CH-N), 65.04 (OCH_2_), 117.23 (S-CH_2_CH=**C**H_2_), 134.82 (S-CH_2_**C**H=CH_2_), 173.91 (-C=O); EIMS: m/z 232.1368 [M + H]^+^, Calcd for C_11_H_22_NO_2_S: 232.1371.

*General procedure for condensation using HBTU*

A solution of 3,4-diacetoxycaffeic acid (**5**) or 3,4-disilylated caffeic acid (**7**) (1 mmol) and triethylamine (4 mmol) in THF (10 mL) was stirred for 15 min. Then, HBTU (1.5 mmol) was added and the resulting mixture was stirred for 10 min. Then, S-allyl cysteine ester (**3a-3e**) (1.2 mmol) was added and the resulting mixture was allowed to stir for 15 h. The solvent was removed under reduced pressure, and the residue was chromatographed on silica gel. Elution with hexane-ethyl acetate (1:1 ratio) afforded compounds **6a-6e** in yields ranging 30-40% [**6a**, 35% (148 mg); **6b**, 39% (170 mg); **6c**, 34% (153 mg); **6d**, 33% (153 mg) and **6e**, 40% (191 mg)] or **8a-8e** in yields ranging 50-60% [**8a**, 50% (283 mg); **8b**, 50% (578 mg); **8c**, 52% (309 mg); 8**d**, 50% (304 mg) and **8e**, 60% (373 mg)].

*Methyl N-{(2E)-3-[3,4-bis(acetyloxy)phenyl]prop-2-enoyl}-S-prop-2-en-1-ylcysteinate (* ***6a*** *)*: M.p. 107-109°C; [α]^25^ + 5.292 (C = 0,019, CHCl_3_); IR (KBr, cm^-1^): ν _max _ 3394 (N-H), 1770, 1743 and 1662 (C=O), 1259 (C-O-C), 1205 ((C=O)-O); ^1^H NMR (CDCl_3_, 300 MHz): δ 2.30 (3H, s, ((**C**H_3_-C=O)-O), 2.31 (3H, s, (**C**H_3_-C=O)-O), 2.95 (1H, dd, *J* = 14.0, 5.0 Hz, S-C**H**_2_CHN), 3.04 (1H, dd, *J* = 14.0, 5.0 Hz, S-C**H**_2_CHN), 3.13 (2H, d, *J* = 7.0 Hz, S-C**H**_2_CH=CH_2_), 3.79 (3H, s, OCH_3_), 4.89-4.98 (1H, m, -C**H**-N), 5.07-5.17 (2H, m, S-CH_2_CH=C**H**_2_), 5.66-5.82 (1H, m, S-CH_2_C**H**=CH_2_), 6.40 (1H, d, *J* = 15.6 Hz, –CO–C**H**=), 6.47 (1H, d, J = 7.5 Hz, -CH-N**H**-C=O), 7.20 (1H, d, *J* = 8.3 Hz, Ar-H), 7.39 (1H, d, *J* = 1.8 Hz, Ar-H), 7.35 (1H, dd, *J* = 8.3, 1.8 Hz, Ar-H), 7.59 (1H, d, *J* = 15.6 Hz, Ar-C**H**=C); ^13^C NMR (CDCl_3_, 75 MHz): δ 20.76 ((**C**H_3_-C=O)-O), 20.79 ((**C**H_3_-C=O)-O), 32.76 (S-**C**H_2_CHN), 35.33 (S-**C**H_2_CH=CH_2_), 52.05 (CH-N), 52.88 (OCH_3_), 118.19 (C=**C**-CO-), 121.08 (S-CH_2_CH=**C**H_2_), 122.61 (Ar), 123.99 (Ar), 126.45 (Ar), 129.16 (Ar), 133.64 (Ar), 133.54 (S-CH_2_**C**H=CH_2_), 140.37 (Ar-**C**=C), 142.50 (Ar-O), 143.29 (Ar-O), 165.09 (-NH-**C**=O), 168.21 ((CH_3_-**C**=O)-O), 168.25 ((CH_3_-**C**=O)-O), 171.44 ((NCH-**C**=O)-O); EIMS: m/z 444.1090 [M + Na]^+^, Calcd for C_20_H_24_NO_7_S: 444.1093

*Ethyl N-{(2E)-3-[3,4-bis(acetyloxy)phenyl]prop-2-enoyl}-S-prop-2-en-1-ylcysteinate (* ***6b*** *)*: M.p. 96-99°C; [α]^25^ + 13.60 (C = 0,835, CHCl_3_); IR (KBr, cm^-1^): ν _max _ 3317 (N-H), 1774, 1734 and 1655 (C=O), 1265 (C-O-C), 1209 ((C=O)-O); ^1^H NMR (CDCl_3_, 600 MHz): δ 1.31 (3H, t, *J* = 7.1 Hz), 2.30 (3H, s, ((**C**H_3_-C=O)-O), 2.31 (3H, s, (**C**H_3_-C=O)-O), 2.96 (1H, dd, *J* = 14.0, 5.4 Hz, S-C**H**_2_CHN), 3.05 (1H, dd, *J* = 14.0, 5.4 Hz, S-C**H**_2_CHN), 3.14 (2H, d, *J* = 7.02 Hz, S-C**H**_2_CH=CH_2_), 4.26 (2H, q, *J* = 7.0 Hz, OCH_2_), 4.90-4.94 (1H, m, -C**H**-N), 5.10-5.15 (2H, m, S-CH_2_CH=C**H**_2_), 5.71-5.80 (1H, m, S-CH_2_C**H**=CH_2_), 6.41 (1H, d, *J* = 15.7 Hz, –CO–C**H**=), 6.51 (1H, d, J = 7.5 Hz, -CH-N**H**-C=O), 7.21 (1H, d, *J* = 8.4 Hz, Ar-H), 7.34 (1H, d, *J* = 2.0 Hz, Ar-H), 7.37 (1H, dd, *J* = 8.4, 2.0 Hz, Ar-H), 7.58 (1H, d, *J* = 15.7 Hz, Ar-C**H**=C); ^13^C NMR (CDCl_3_, 125 MHz): δ 14.28 (CH_3_), 20.76 ((**C**H_3_-C=O)-O), 20.79 ((**C**H_3_-C=O)-O), 32.89 (S-**C**H_2_CHN), 35.39 (S-**C**H_2_CH=CH_2_), 52.16 (CH-N), 62.11 (OCH_2_), 118.03 (S-CH_2_CH=**C**H_2_), 118.13 (C=**C**-CO-), 121.16 (Ar), 122.61 (Ar), 123.99 (Ar), 126.44 (Ar), 133.73 (S-CH_2_**C**H=CH_2_), 140.30 (Ar-**C**=C), 142.50 (Ar-O), 143.29 (Ar-O), 165.08 (-NH-**C**=O), 168.20 ((CH_3_-**C**=O)-O), 168.26 ((CH_3_-**C**=O)-O), 170.94 ((NCH-**C**=O)-O); EIMS: m/z 436.1430 [M + H]^+^, Calcd for C_21_H_26_NO_7_S: 436.1431.

*Propyl N-{(2E)-3-[3,4-bis(acetyloxy)phenyl]prop-2-enoyl}-S-prop-2-en-1-ylcysteinate (* ***6c*** *)*: M.p. 80-82°C; [α]^25^ + 1.442 (C = 0,011, CHCl_3_); IR (KBr, cm^-1^): ν _max _ 3315 (N-H), 1766, 1735 and 1658 (C=O), 1209 (C-O-C), 1184 ((C=O)-O); ^1^H NMR (CDCl_3_, 300 MHz): δ 0.96 (3H, t, *J* = 7.5 Hz), 1.62-1.77 (2H, m), 2.29 (3H, s, ((CH_3_-C=O)-O), 2.30 (3H, s, (CH_3_-C=O)-O), 2.94 (1H, dd, *J* = 13.9, 5.3 Hz, S-C**H**_2_CHN), 3.04 (1H, dd, *J* = 13.9, 4.9 Hz, S-C**H**_2_CHN), 3.13 (2H, d, *J* = 7.20 Hz, S-C**H**_2_CH=CH_2_), 4.14 (2H, t, *J* = 6.7 Hz, OCH_2_), 4.87-4.97 (1H, m, -CH-N), 5.05-5.18 (2H, m, S-CH_2_CH=C**H**_2_), 5.64-5.82 (1H, m, S-CH_2_C**H**=CH_2_), 6.41 (1H, d, *J* = 15.6 Hz, –CO–C**H**=), 6.52 (1H, d, J = 7.5 Hz, -CH-N**H**-C=O), 7.20 (1H, d, *J* = 8.3 Hz, Ar-H), 7.24 (1H, d, *J* = 1.6 Hz, Ar-H), 7.34 (1H, dd, *J* = 8.3, 1.6 Hz, Ar-H), 7.58 (1H, d, *J* = 15.6 Hz, Ar-C**H**=C); ^13^C NMR (CDCl_3_, 75 MHz): δ 10.46 (CH_3_), 20.77 ((**C**H_3_-C=O)-O), 22.00 ((**C**H_3_-C=O)-O), 32.86 (S-**C**H_2_CHN), 35.37 (S-**C**H_2_CH=CH_2_), 52.16 (CH-N), 67.61 (OCH_2_), 118.12 (S-CH_2_CH=**C**H_2_), 121.16 (=**C**-CO-), 122.58 (Ar), 123.97 (Ar), 126.44 (Ar), 129.14 (Ar), 133.66 (S-CH_2_**C**H=CH_2_), 140.25 (Ar-C=), 142.48 (Ar-O), 143.25 (Ar-O), 165.09 (N-C=O), 168.19 ((CH_3_-**C**=O)-O), 168.24 ((CH_3_-**C**=O)-O), 171.62 ((NCH-**C**=O)-O); EIMS: m/z 472.1401 [M + Na]^+^, Calcd for C_22_H_27_NO_7_S-Na: 472.1406.

*Butyl N-{(2E)-3-[3,4-bis(acetyloxy)phenyl]prop-2-enoyl}-S-prop-2-en-1-ylcysteinate (* ***6d*** *): *M.p. 94-97°C; [α]^25^ + 8.30 (C = 0,65, CHCl_3_); IR (KBr, cm^-1^): ν _max _ 3309 (N-H), 1774, 1742 and 1662 (C=O), 1265 (C-O-C), 1209 ((C=O)-O); ^1^H NMR (CDCl_3_, 600 MHz): δ 0.95 (3H, t, *J* = 7.2 Hz), 1.36-1.44 (2H, m), 1.61-1.72 (2H, m), 2.30 (3H, s, ((CH_3_-C=O)-O), 2.31 (3H, s, ((CH_3_-C=O)-O), 2.95 (1H, dd, *J* = 13.8, 5.4 Hz, S-C**H**_2_CHN), 3.05 (1H, dd, *J* = 13.9, 5.4 Hz, S-C**H**_2_CHN), 3.10-3.16 (2H, m, S-C**H**_2_CH=CH_2_), 4.19 (2H, q, *J* = 6.9 Hz, OCH_2_), 4.90-4.96 (1H, m, -CH-N), 5.08-5.15 (2H, m, S-CH_2_CH=C**H**_2_), 5.70-5.79 (1H, m, S-CH_2_C**H**=CH_2_), 6.41 (1H, d, *J* = 15.6 Hz, –CO–C**H**=), 6.50 (1H, d, J = 7.2 Hz, -CH-N**H**-C=O), 7.21 (1H, d, *J* = 8.3 Hz, Ar-H), 7.35 (1H, s, Ar-H), 7.38 (1H, d, *J* = 8.3, Ar-H), 7.59 (1H, d, *J* = 15.6 Hz, Ar-C**H**=C); ^13^C NMR (CDCl_3_, 125 MHz): δ 13.77 (CH_3_), 19.19 (CH_2_), 20.70 ((**C**H_3_-C=O)-O), 20.78 ((**C**H_3_-C=O)-O), 23.18 (CH_2_), 32.97 (S-**C**H_2_CHN), 35.41 (S-**C**H_2_CH=CH_2_), 52.20 (CH-N), 65.94 (OCH_2_), 118.01 (S-CH_2_CH=**C**H_2_), 118.11 (=**C**-CO-), 121.17 (Ar), 122.60 (Ar), 123.98 (Ar), 126.43 (Ar), 133.73 (S-CH_2_**C**H=CH_2_), 140.29 (Ar-C=), 142.51 (Ar-O), 143.29 (Ar-O), 165.09 (N-C=O), 168.18 ((CH_3_-**C**=O)-O), 168.23 ((CH_3_-**C**=O)-O), 171.02 ((NCH-**C**=O)-O); EIMS: m/z 464.1743 [M + H]^+^, Calcd for C_23_H_29_NO_7_S: 464.1745.

*Pentyl N-{(2E)-3-[3,4-bis(acetyloxy)phenyl]prop-2-enoyl}-S-prop-2-en-1-ylcysteinate (* ***6e*** *): *M.p. 101-103°C; [α]^25^ + 3.087 (C = 0,0119, CHCl_3_); IR (KBr, cm^-1^): ν _max _ 3317 (N-H), 1768, 1743 and 1656 (C=O), 1219 (C-O-C), 1184 ((C=O)-O); ^1^H NMR (CDCl_3_, 300 MHz): δ 0.90 (3H, t, *J* = 7.1 Hz), 1.28-1.39 (4H, m), 1.60-1.74 (2H, m), 2.29 (3H, s, ((CH_3_-C=O)-O), 2.30 (3H, s, ((CH_3_-C=O)-O), 2.94 (1H, dd, *J* = 13.9, 5.3 Hz, S-C**H**_2_CHN), 3.05 (1H, dd, *J* = 13.9, 4.8 Hz, S-C**H**_2_CHN), 3.13 (2H, d, *J* = 7.3 Hz, S-C**H**_2_CH=CH_2_), 4.18 (2H, t, *J* = 6.8 Hz, OCH_2_), 4.87-4.96 (1H, m, -C**H**-N), 5.06-5.10 (2H, m, S-CH_2_CH=C**H**_2_), 5.66-5.82 (1H, m, S-CH_2_C**H**=CH_2_), 6.40 (1H, d, *J* = 15.6 Hz, –CO–C**H**=), 6.48 (1H, d, J = 7.4 Hz, -CH-N**H**-C=O), 7.20 (1H, d, *J* = 8.4 Hz, Ar-H), 7.34 (1H, d, *J* = 1.8 Hz, Ar-H), 7.38 (1H, dd, *J* = 8.4, 1.8 Hz, Ar-H), 7.58 (1H, d, *J* = 15.6 Hz, Ar-C**H**=C); ^13^C NMR (CDCl_3_, 75 MHz): δ 14.07 (CH_3_), 20.77 ((**C**H_3_-C=O)-O), 20.80 ((**C**H_3_-C=O)-O), 22.37 (CH_2_), 28.07 (CH_2_), 28.29 (CH_2_), 32.89 (S-**C**H_2_CHN), 35.41 (S-**C**H_2_CH=CH_2_), 52.18 (CH-N), 66.23 (OCH_2_), 118.13 (S-CH_2_CH=**C**H_2_), 119.0 (=**C**-CO-), 121.15 (Ar), 122.60 (Ar), 123.99 (Ar), 126.44 (Ar), 133.67 (S-CH_2_**C**H=CH_2_), 140.29 (Ar-**C**=C), 142.50 (Ar-O), 143.28 (Ar-O), 165.07 (N-C=O), 168.20 ((CH_3_-**C**=O)-O), 168.25 ((CH_3_-**C**=O)-O), 171.02 ((NCH-**C**=O)-O); EIMS: m/z 500.1719 [M + Na]^+^, Calcd for C_24_H_31_NO_7_S-Na: 500.1719.

*Methyl N-[(2E)-3-(3,4-bis{[tert-butyl(dimethyl)silyl]oxy}phenyl)prop-2-enoyl]-S-prop-2-en-1-yl-L-cysteinate* *(****8a****)*: colorless oil; [α]^25^ + 2.50 (C = 0,50, CHCl_3_); IR (KBr, cm^-1^): ν _max _ 3309 (N-H), 1742 and 1666 (C=O), 1424 (Si-C), 1249 (C-O-C), 1202 ((C=O)-O), 1106 (Si-O); ^1^H NMR (CDCl_3_, 600 MHz): δ 0.21 (12H, s, -Si-C**H**_3_), 0.98 (9H, s, -C-(C**H**_3_)_3_), 1.0 (9H, s, -C-(C**H**_3_)_3_), 2.96 (1H, dd, *J* = 14.0, 5.0 Hz, S-C**H**_2_CHN), 3.04 (1H, dd, *J* = 14.0, 5.0 Hz, S-C**H**_2_CHN), 3.14 (2H, t_app_, *J* = 7.5 Hz, S-C**H**_2_CH=CH_2_), 3.80 (3H, s, OCH_3_), 4.93-4.98 (1H, m, -C**H**-N), 5.08-5.16 (2H, m, S-CH_2_CH=C**H**_2_), 5.70-5.80 (1H, m, S-CH_2_C**H**=CH_2_), 6.25 (1H, d, *J* = 15.5 Hz, –CO–C**H**=C), 6.35 (1H, d, J = 7.2 Hz, -CH-N**H**-C=O), 6.81 (1H, d, *J* = 8.0 Hz, Ar-H), 6.98-7.02 (2H, m, Ar-H), 7.52 (1H, d, *J* = 15.5 Hz, Ar-C**H**=C); ^13^C NMR (CDCl_3_, 125 MHz): δ -3.92 (-Si-**C**H_3_), -3.44 (-Si-**C**H_3_), 18.60 (-Si-**C**-(CH_3_)_3_), 18.64 (-Si-**C**-(CH_3_)_3_), 26.03 (-C-(**C**H_3_)_3_), 26.07 (-C-(**C**H_3_)_3_), 32.96 (S-**C**H_2_CHN), 35.40 (S-**C**H_2_CH=CH_2_), 52.04 (CH-N), 52.85 (OCH_2_), 117.58 (Ar), 118.07 (S-CH_2_CH=**C**H_2_), 118.16 (Ar), 120.88 (C=**C**-CO-), 121.29 (Ar), 122.01 (Ar), 133.73 (S-CH_2_**C**H=CH_2_), 142.07 (Ar-**C**=C), 147.27 (Ar-O), 149.23 (Ar-O), 165.90 (-NH-**C**=O), 171.67 (CH-**C**=O)-O). 

*EIMS: m/z *566, 2751 [M + H]^+^*, *Calcd for C_28_H_48_NO_5_SSi_2_: 566.1994.

*Ethyl N-[(2E)-3-(3,4-bis{[tert-butyl(dimethyl)silyl]oxy}phenyl)prop-2-enoyl]-S-prop-2-en-1-yl-L-cysteinate* *(****8b****)*: colorless oil; [α]^25^ + 2.30 (C = 0,585, CHCl_3_); IR (KBr, cm^-1^): ν _max _ 3293 (N-H), 1750 and 1662 (C=O), Si-C (1416), 1257 (C-O-C), 1209 ((C=O)-O), 1209 (Si-O); ^1^H NMR (CDCl_3_, 600 MHz): δ 0.21 (12H, s, -Si-C**H**_3_), 0.98 (9H, s, -C-(C**H**_3_)_3_), 1.0 (9H, s, -C-(C**H**_3_)_3_), 1.31 (3H, t, *J* = 7.2 Hz), 2.96 (1H, dd, *J* = 13.94, 5.2 Hz, S-C**H**_2_CHN), 3.05 (1H, dd, *J* = 13.94, 5.2 Hz, S-C**H**_2_CHN), 3.14 (2H, t_app_, *J* = 6.8 Hz, S-C**H**_2_CH=CH_2_), 4.26 (2H, q, J = 7.1 Hz, OCH_2_), 4.91-4.96 (1H, m, -C**H**-N), 5.09-5.16 (2H, m, S-CH_2_CH=C**H**_2_), 5.70-5.79 (1H, m, S-CH_2_C**H**=CH_2_), 6.26 (1H, d, *J* = 15.6 Hz, –CO–C**H**=C), 6.36 (1H, d, J = 7.5 Hz, -CH-N**H**-C=O), 6.81 (1H, d, *J* = 8.0 Hz, Ar-H), 6.98-7.02 (2H, m, Ar-H), 7.52 (1H, d, *J* = 15.6 Hz, Ar-C**H**=C); ^13^C NMR (CDCl_3_, 125 MHz): δ -3.92 (-Si-**C**H_3_), -3.43 (-Si-**C**H_3_), 14.31 (-Si-**C**-(CH_3_)_3_), 18.60 (-Si-**C**-(CH_3_)_3_), 18.64 (-Si-**C**-(CH_3_)_3_), 26.03 (-C-(**C**H_3_)_3_), 26.07 (-C-(**C**H_3_)_3_), 33.03 (S-**C**H_2_CHN), 35.45 (S-**C**H_2_CH=CH_2_), 52.15 (CH-N), 62.06 (OCH_2_), 117.54 (Ar), 118.09 (S-CH_2_CH=**C**H_2_), 120.62 (Ar), 121.29 ((C=**C**-CO-), 121.99 (Ar), 128.32 (Ar), 133.77 (S-CH_2_**C**H=CH_2_), 138.59 (Ar-**C**=C), 147.27 (Ar-O), 149.60 (Ar-O), 165.88 (-NH-**C**=O), 171.17 (CH-**C**=O)-O). *EIMS: m/z *580,2944 [M + H]^+^*, *Calcd for C_29_H_50_NO_5_SSi_2_: 580.2170.

*Propyl N-[(2E)-3-(3,4-bis{[tert-butyl(dimethyl)silyl]oxy}phenyl)prop-2-enoyl]-S-prop-2-en-1-yl-L-cysteinate* *(****8c****)*: colorless oil; [α]^25^ + 1.70 (C = 0,665, CHCl_3_); IR (KBr, cm^-1^): ν _max _ 3277 (N-H), 1742 and 1662 (C=O), 1416 (Si-C), 1257 (C-O-C), 1209 ((C=O)-O), 1122 (Si-O); ^1^H NMR (CDCl_3_, 600 MHz): δ 0.21 (12H, s, -Si-C**H**_3_), 0.98 (3H, t, *J* = 7.0 Hz), 0.99 (9H, s, -C-(C**H**_3_)_3_), 1.0 (9H, s, -C-(C**H**_3_)_3_), 1.67-1.74 (2H, m), 2.95 (1H, dd, *J* = 14.0, 5.3 Hz, S-C**H**_2_CHN), 3.05 (1H, dd, *J* = 14.0, 5.3 Hz, S-C**H**_2_CHN), 3.15 (2H, t_app_, *J* = 6.8 Hz, S-C**H**_2_CH=CH_2_), 4.15 (2H, t, *J* = 6.6 Hz, OCH_2_), 4.92-4.97 (1H, m, -C**H**-N), 5.10-5.15 (2H, m, S-CH_2_CH=C**H**_2_), 5.71-5.79 (1H, m, S-CH_2_C**H**=CH_2_), 6.25 (1H, d, *J* = 15.5 Hz, –CO–C**H**=C), 6.37 (1H, d, J = 7.6 Hz, -CH-N**H**-C=O), 6.81 (1H, d, *J* = 8.0 Hz, Ar-H), 6.98-7.02 (2H, m, Ar-H), 7.52 (1H, d, *J* = 15.5 Hz, Ar-C**H**=C); ^13^C NMR (CDCl_3_, 125 MHz): δ -4.06 (-Si-**C**H_3_), -3.92 (-Si-**C**H_3_), 10.50 (-Si-**C**-(CH_3_)_3_), 18.60 (-Si-**C**-(CH_3_)_3_), 18.64 (-Si-**C**-(CH_3_)_3_), 22.05 (CH_2_), 26.03 (-C-(**C**H_3_)_3_), 26.06 (-C-(**C**H_3_)_3_), 33.09 (S-**C**H_2_CHN), 35.46 (S-**C**H_2_CH=CH_2_), 52.16 (CH-N), 67.60 (OCH_2_), 117.17 (Ar), 118.10 (S-CH_2_CH=**C**H_2_), 120.62 (Ar), 121.29 ((C=**C**-CO-), 121.98 (Ar), 128.32 (Ar), 133.76 (S-CH_2_**C**H=CH_2_), 142.05 (Ar-**C**=C), 147.26 (Ar-O), 149.19 (Ar-O), 165.90 (-NH-**C**=O), 171.27 (CH-**C**=O)-O). *EIMS: m/z *594, 3136 [M + H]^+^*, *Calcd for C_30_H_52_NO_5_SSi_2_: 594.2331.

*Butyl N-[(2E)-3-(3,4-bis{[tert-butyl(dimethyl)silyl]oxy}phenyl)prop-2-enoyl]-S-prop-2-en-1-yl-L-cysteinate* *(****8d****)*: colorless oil; [α]^25^ + 2.50 (C = 0,64, CHCl_3_); IR (KBr, cm^-1^): ν _max _ 3285 (N-H), 1742 and 1655 (C=O), 1416 (Si-C), 1265 (C-O-C), 1209 ((C=O)-O), 1122 (Si-O); ^1^H NMR (CDCl_3_, 600 MHz): δ 0.21 (12H, s, -Si-C**H**_3_), 0.95 (3H, t, *J* = 7.1 Hz), 0.98 (9H, s, -C-(C**H**_3_)_3_), 1.0 (9H, s, -C-(C**H**_3_)_3_), 1.34-1.45 (2H, m), 1.61-1.71 (2H, m), 2.95 (1H, dd, *J* = 13.90, 5.2 Hz, S-C**H**_2_CHN), 3.05 (1H, dd, *J* = 13.90, 5.4 Hz, S-C**H**_2_CHN), 3.05 (2H, t_app_, *J* = 6.7 Hz, S-C**H**_2_CH=CH_2_), 4.20 (2H, t, *J* = 6.7 Hz, OCH_2_), 4.91-4.96 (1H, m, -C**H**-N), 5.08-5.016 (2H, m, S-CH_2_CH=C**H**_2_), 5.70-5.79 (1H, m, S-CH_2_C**H**=CH_2_), 6.25 (1H, d, *J* = 15.5 Hz, –CO–C**H**=C), 6.36 (1H, d, J = 7.6 Hz, -CH-N**H**-C=O), 6.81 (1H, d, *J* = 8.0 Hz, Ar-H), 6.97-7.02 (2H, m, Ar-H), 7.52 (1H, d, *J* = 15.5 Hz, Ar-C**H**=C); ^13^C NMR (CDCl_3_, 125 MHz): δ -4.02 (-Si-**C**H_3_), -3.92 (-Si-**C**H_3_), 13.80 (-Si-**C**-(CH_3_)_3_), 18.60 (-Si-**C**-(CH_3_)_3_), 18.64 (-Si-**C**-(CH_3_)_3_), 19.22 (CH_2_), 26.03 (-C-(**C**H_3_)_3_), 26.07 (-C-(**C**H_3_)_3_), 30.65 (CH_2_), 33.10 (S-**C**H_2_CHN), 35.47 (S-**C**H_2_CH=CH_2_), 52.17 (CH-N), 65.90 (OCH_2_), 117.68 (Ar), 118.09 (S-CH_2_CH=**C**H_2_), 120.62 (Ar), 121.99 ((C=**C**-CO-), 123.62 (Ar), 128.32 (Ar), 133.77 (S-CH_2_**C**H=CH_2_), 142.05 (Ar-**C**=C), 147.27 (Ar-O), 149.20 (Ar-O), 165.90 (-NH-**C**=O), 171.26 (CH-**C**=O)-O). *EIMS: m/z *609.3315 [M + H]^+^*, *Calcd for C_31_H_54_NO_5_SSi_2_: 609.3293.

*Pentyl N-[(2E)-3-(3,4-bis{[tert-butyl(dimethyl)silyl]oxy}phenyl)prop-2-enoyl]-S-prop-2-en-1-yl-L-cysteinate* *(****8f****)*: colorless oil; [α]^25^ + 2.80 (C = 0,925, CHCl_3_); IR (KBr, cm^-1^): ν _max _ 3277 (N-H), 1750 and 1662 (C=O), 1416 (Si-C), 1249 (C-O-C), 1209 ((C=O)-O), 1122 (Si-O); ^1^H NMR (CDCl_3_, 600 MHz): δ 0.21 (12H, s, -Si-C**H**_3_), 0.91 (3H, t, *J* = 7.0 Hz), 0.98 (9H, s, -C-(C**H**_3_)_3_), 1.0 (9H, s, -C-(C**H**_3_)_3_), 1.29-1.39 (4H, m), 1.60-1.73 (2H, m), 2.95 (1H, dd, *J* = 13.90, 5.2 Hz, S-C**H**_2_CHN), 3.05 (1H, dd, *J* = 13.90, 5.4 Hz, S-C**H**_2_CHN), 3.15 (2H, t_app_, *J* = 6.9 Hz, S-C**H**_2_CH=CH_2_), 4.18 (2H, t, *J* = 6.6 Hz, OCH_2_), 4.91-4.97 (1H, m, -C**H**-N), 5.10-5.16 (2H, m, S-CH_2_CH=C**H**_2_), 5.70-5.80 (1H, m, S-CH_2_C**H**=CH_2_), 6.24 (1H, d, *J* = 15.7 Hz, –CO–C**H**=C), 6.37 (1H, d, J = 7.5 Hz, -CH-N**H**-C=O), 6.81 (1H, d, *J* = 8.0 Hz, Ar-H), 6.98-7.02 (2H, m, Ar-H), 7.52 (1H, d, *J* = 15.7 Hz, Ar-C**H**=C); ^13^C NMR (CDCl_3_, 125 MHz): δ -4.02 (-Si-**C**H_3_), -3.92 (-Si-**C**H_3_), 14.09 (-Si-**C**-(CH_3_)_3_), 18.60 (-Si-**C**-(CH_3_)_3_), 18.64 (-Si-**C**-(CH_3_)_3_), 22.40 (CH_2_), 26.03 (-C-(**C**H_3_)_3_), 26.06 (-C-(**C**H_3_)_3_), 28.12 (CH_2_), 28.30 (CH_2_), 33.09 (S-**C**H_2_CHN), 35.46 (S-**C**H_2_CH=CH_2_), 52.17 (CH-N), 66.18 (OCH_2_), 117.68 (Ar), 118.08 (S-CH_2_CH=**C**H_2_), 120.61 (Ar), 121.29 ((C=**C**-CO-), 121.98 (Ar), 128.32 (Ar), 133.77 (S-CH_2_**C**H=CH_2_), 142.04 (Ar-**C**=C), 147.26 (Ar-O), 149.19 (Ar-O), 165.88 (-NH-**C**=O), 171.25 (CH-**C**=O)-O). *EIMS: m/z *623,3478 [M + H]^+^*, *Calcd for C_32_H_56_NO_5_SSi_2_: 623.3431.

*Procedure for desprotection of compounds 8a-8e*

To a solution of compound 8 (1 mmol) in THF-H_2_O (1:1) (10 mL) was added KF (4 mmol) and the mixture was stirred for 12 h. Then an aqueous saturated solution of NH_4_Cl was added and the mixture was extracted with dichloromethane (3 × 10 mL). The combined organic phases were dried over anhydrous MgSO_4_ and the solvent was evaporated under reduced pressure to afford hybrids **9a-9e** in yields ranging 50%-96% [**9a**, 69% (233 mg); **9b**, 50% (176 mg); **9c**, 67% (246 mg); **9d**, 86% (326 mg) and **9e**, 96% (378 

mg)].

*Methyl N-[(2E)-3-(3,4-dihydroxyphenyl)-2-propenoyl]-S-2-propen-1-yl-L-cysteinate (* ***9a*** *)*: M.p. 101-103°C; [α]^25^ + 10.10 (C = 0,60, CHCl_3_); IR (KBr, cm^-1^): ν _max _ 3460 (OH), 3222 (N-H), 1750 and 1665 (C=O), 1265 (C-O-C), 1209 ((C=O)-O); ^1^H NMR (CDCl_3_, 600 MHz): δ 2.93 (1H, dd, *J* = 14.0, 5.0 Hz, S-C**H**_2_CHN), 3.02 (1H, dd, *J* = 14.10, 5.0 Hz, S-C**H**_2_CHN), 3.12 (2H, t_app_, *J* = 7.0 Hz, S-C**H**_2_CH=CH_2_), 3.78 (s, OCH_3_), 4.86-4.96 (1H, m, -C**H**-N), 5.04-5.16 (2H, m, S-CH_2_CH=C**H**_2_), 5.67-5.78 (1H, m, S-CH_2_C**H**=CH_2_), 6.26 (1H, d, *J* = 15.7 Hz, –CO–C**H**=C), 6.78 (d, *J* = 7.2 Hz, -CH-N**H**-C=O), 6.81 (1H, d, J = 8.0 Hz, Ar), 6.86 (1H, dd, J = 8.0, 1.5 Hz, Ar), 7.02 (1H, d, J = 1.5 Hz, Ar), 7.47 (1H, d, *J* = 15.6 Hz, Ar-C**H**=C); ^13^C NMR (CDCl_3_, 125 MHz): δ 32.65 (S-**C**H_2_CHN), 35.26 (S-**C**H_2_CH=CH_2_), 52.22 (CH-N), 68.13 (OCH_3_), 114.55 (Ar), 115.54 (Ar), 116.62 ((C=**C**-CO-), 118.27 (S-CH_2_CH=**C**H_2_), 121.98 (Ar), 127.16 (Ar), 133.59 (S-CH_2_**C**H=CH_2_), 143.13 (Ar-**C**=C), 144.39 (Ar-O), 146.96 (Ar-O), 167.18 (-NH-**C**=O), 171.83 (CH-**C**=O)-O); EIMS: m/z 338.1062 [M + H]^+^, Calcd for C_16_H_20_NO_5_S: 338.1061.

*Ethyl N-[(2E)-3-(3,4-dihydroxyphenyl)-2-propenoyl]-S-2-propen-1-yl-L-cysteinate (* ***9b*** *)*: M.p. 110-112°C; [α]^25^ + 12.70 (C = 0,54, CHCl_3_); IR (KBr, cm^-1^): ν _max _ 3467 (OH), 3213 (N-H), 1742 and 1662 (C=O), 1265 (C-O-C), 1202 ((C=O)-O); ^1^H NMR (CDCl_3_, 600 MHz): δ 1.29 (3H, t, *J* = 7.0 Hz), 2.93 (1H, dd, *J* = 14.10, 5.2 Hz, S-C**H**_2_CHN), 3.03 (1H, dd, *J* = 14.10, 5.2 Hz, S-C**H**_2_CHN), 3.13 (2H, t_app_, *J* = 6.2 Hz, S-C**H**_2_CH=CH_2_), 4.24 (2H, t, *J* = 7.0 Hz, OCH_2_), 4.86-4.93 (1H, m, -C**H**-N), 5.07-5.13 (2H, m, S-CH_2_CH=C**H**_2_), 5.67-5.77 (1H, m, S-CH_2_C**H**=CH_2_), 6.26 (1H, d, *J* = 15.6 Hz, –CO–C**H**=C), 6.77-6.87 (3H, m, Ar-H, -CH-N**H**-C=O), 7.01 (1H, s, Ar-H), 7.46 (1H, d, *J* = 15.6 Hz, Ar-C**H**=C); ^13^C NMR (CDCl_3_, 125 MHz): δ 14.24 (CH_3_), 32.66 (S-**C**H_2_CHN), 35.28 (S-**C**H_2_CH=CH_2_), 52.34 (CH-N), 62.41 (OCH_2_), 114.76 (Ar), 115.57 (Ar), 116.57 ((C=**C**-CO-), 118.25 (S-CH_2_CH=**C**H_2_), 121.95 (Ar), 127.12 (Ar), 133.59 (S-CH_2_**C**H=CH_2_), 143.18 (Ar-**C**=C), 144.36 (Ar-O), 147.0 (Ar-O), 167.31 (-NH-**C**=O), 171.40 (CH-**C**=O)-O); EIMS: m/z 352.1219 [M + H]^+^, Calcd for C_17_H_22_NO_5_S: 352.1215.

*Propyl N-[(2E)-3-(3,4-dihydroxyphenyl)-2-propenoyl]-S-2-propen-1-yl-L-cysteinate (* ***9c*** *)*: M.p. 129-131°C; [α]^25^ + 12.70 (C = 0,61, CHCl_3_); IR (KBr, cm^-1^): ν _max _ 3467 (OH), 3206 (N-H), 1750 and 1662 (C=O), 1257 (C-O-C), 1209 ((C=O)-O); ^1^H NMR (CDCl_3_, 600 MHz): δ 0.95 (3H, t, *J* = 7.0 Hz), 1.63-1.73 (CH_2_, m), 2.94 (1H, dd, *J* = 14.10, 5.2 Hz, S-C**H**_2_CHN), 3.03 (1H, dd, *J* = 14.10, 5.2 Hz, S-C**H**_2_CHN), 3.13 (2H, t_app_, *J* = 6.2 Hz, S-C**H**_2_CH=CH_2_), 4.15 (2H, q, *J* = 7.0 Hz, OCH_2_), 4.89-4.94 (1H, m, -C**H**-N), 5.08-5.14 (2H, m, S-CH_2_CH=C**H**_2_), 5.69-5.77 (1H, m, S-CH_2_C**H**=CH_2_), 6.27 (1H, d, *J* = 15.6 Hz, –CO–C**H**=C), 6.76 (d, *J* = 7.2 Hz, -CH-N**H**-C=O), 6.82 (1H, d, J = 8.0 Hz, Ar), 6.86 (1H, d, *J* = 8.0 Hz, Ar), 7.02 (1H, s, Ar), 7.48 (1H, d, *J* = 15.5 Hz, Ar-C**H**=C); ^13^C NMR (CDCl_3_, 125 MHz): δ 10.48 (CH_3_), 22.0 (CH_2_), 32.77 (S-**C**H_2_CHN), 35.32 (S-**C**H_2_CH=CH_2_), 52.31 (CH-N), 67.90 (OCH_2_), 114.74 (Ar), 115.50 (Ar), 116.63 ((C=**C**-CO-), 118.24 (S-CH_2_CH=**C**H_2_), 121.86 (Ar), 127.15 (Ar), 133.60 (S-CH_2_**C**H=CH_2_), 143.13 (Ar-**C**=C), 144.33 (Ar-O), 146.97 (Ar-O), 167.15 (-NH-**C**=O), 171.45 (CH-**C**=O)-O); EIMS: m/z 366.1375 [M + H]^+^, Calcd for C_18_H_24_NO_5_S: 366.1377.

*Butyl N-[(2E)-3-(3,4-dihydroxyphenyl)-2-propenoyl]-S-2-propen-1-yl-L-cysteinate (* ***9d*** *)*: M.p. 119-121°C; [α]^25^ + 11.0 (C = 0,65, CHCl_3_); IR (KBr, cm^-1^): ν _max _ 3467 (OH), 3206 (N-H), 1734 and 1662 (C=O), 1265 (C-O-C), 1209 ((C=O)-O); ^1^H NMR (CDCl_3_, 600 MHz): δ 0.93 (3H, t, *J* = 7.0 Hz), 1.32-1.46 (CH_2_, m), 1.57-1.71 (CH_2_, m), 2.94 (1H, dd, *J* = 14.10, 5.2 Hz, S-C**H**_2_CHN), 3.03 (1H, dd, *J* = 14.10, 5.2 Hz, S-C**H**_2_CHN), 3.13 (2H, t_app_, *J* = 6.2 Hz, S-C**H**_2_CH=CH_2_), 4.19 (2H, q, *J* = 6.2 Hz, OCH_2_), 4.88-4.94 (1H, m, -C**H**-N), 5.07-5.15 (2H, m, S-CH_2_CH=C**H**_2_), 5.66-5.80 (1H, m, S-CH_2_C**H**=CH_2_), 6.27 (1H, d, *J* = 15.6 Hz, –CO–C**H**=C), 6.73 (d, *J* = 7.2 Hz, -CH-N**H**-C=O), 6.83 (1H, d, J = 8.2 Hz, Ar), 6.88 (1H, d, *J* = 8.2 Hz, Ar), 7.04 (1H, s, Ar), 7.49 (1H, d, *J* = 15.5 Hz, Ar-C**H**=C); ^13^C NMR (CDCl_3_, 125 MHz): δ 13.77 (CH_3_), 19.19 (CH_2_), 30.60 (CH_2_), 32.80 (S-**C**H_2_CHN), 35.34 (S-**C**H_2_CH=CH_2_), 52.33 (CH-N), 66.19 (OCH_2_), 114.77 (Ar), 115.52 (Ar), 116.70 ((C=**C**-CO-), 118.22 (S-CH_2_CH=**C**H_2_), 121.84 (Ar), 127.19 (Ar), 133.63 (S-CH_2_**C**H=CH_2_), 143.11 (Ar-**C**=C), 144.38 (Ar-O), 146.96 (Ar-O), 167.10 (-NH-**C**=O), 171.41 (CH-**C**=O)-O); EIMS: m/z 380.1532 [M + H]^+^, Calcd for C_19_H_26_NO_5_S: 380.1536.

*Pentyl N-[(2E)-3-(3,4-dihydroxyphenyl)-2-propenoyl]-S-2-propen-1-yl-L-cysteinate (* ***9e*** *)*: M.p. 111-113°C; [α]^25^ + 14.4 (C = 0,80, CHCl_3_); IR (KBr, cm^-1^): ν _max _ 3460 (OH), 3206 (N-H), 1734 and 1655 (C=O), 1265 (C-O-C), 1209 ((C=O)-O); ^1^H NMR (CDCl_3_, 600 MHz): δ 0.89 (3H, t, *J* = 6.5 Hz), 1.28-1.37 (CH_2_, m), 1.61-1.69 (CH_2_, m), 2.92 (1H, dd, *J* = 14.10, 5.2 Hz, S-C**H**_2_CHN), 3.03 (1H, dd, *J* = 14.10, 5.2 Hz, S-C**H**_2_CHN), 3.13 (2H, t_app_, *J* = 6.2 Hz, S-C**H**_2_CH=CH_2_), 4.17 (2H, q, *J* = 6.2 Hz, OCH_2_), 4.87-4.94 (1H, m, -C**H**-N), 5.07-5.14 (2H, m, S-CH_2_CH=C**H**_2_), 5.68-5.77 (1H, m, S-CH_2_C**H**=CH_2_), 6.25 (1H, d, *J* = 15.6 Hz, –CO–C**H**=C), 6.78-6.87 (3H, m), 7.01 (1H, s, Ar), 7.46 (1H, d, *J* = 15.5 Hz, Ar-C**H**=C); ^13^C NMR (CDCl_3_, 125 MHz): δ 14.06 (CH_3_), 18.12 (CH_2_), 21.20 (CH_2_), 28.06 (CH_2_), 32.74 (S-**C**H_2_CHN), 35.29 (S-**C**H_2_CH=CH_2_), 52.31 (CH-N), 66.47 (OCH_2_), 114.54 (Ar), 115.47 (Ar), 116.59 ((C=**C**-CO-), 118.21 (S-CH_2_CH=**C**H_2_), 121.98 (Ar), 127.13 (Ar), 133.60 (S-CH_2_**C**H=CH_2_), 143.11 (Ar-**C**=C), 144.40 (Ar-O), 146.99 (Ar-O), 167.18 (-NH-**C**=O), 171.55 (CH-**C**=O)-O); EIMS: m/z 394.1688 [M + H]^+^, Calcd for C_20_H_28_NO_5_S: 394.1691.

***4.2.*** *Biological activity assays*

*Cell lines and culture medium*

Biological assays were performed using an adenocarcinoma colon cancer cell line (SW480) and non-malignant cells (CHO-K1). These were obtained from the European Collection of Authenticated Cell Cultures (ECACC, England) and maintained in Dulbecco’s Modified Eagle Medium, supplemented with 10% heat-inactivated (56 °C) horse serum, 1% penicillin/streptomycin and 1% non-essential amino acids (Gibco Invitrogen, Carlsbad, USA). For all experiments, horse serum was reduced to 3%, and the medium was supplemented with 5 mg/ml transferrin, 5 ng/mL selenium and 10 mg/ml insulin (ITS-defined medium; Gibco, Invitrogen, Carlsbad, USA) (32). 

*Cell Viability*

The cell viability of the synthesized hybrids, lead, and reference compounds was evaluated through Sulforhodamine B (SRB) assay, a colorimetric test that is based on staining of total cellular protein of adherent cells. The cells were seeded to a ﬁnal density of 20.000 cells/well in 96-well tissue culture plates and incubated at 37 °C in a humidified atmosphere at 5% CO_2_. All cultures were allowed to grow for 24 h and afterward they were treated with DMSO (dimethylsulfoxide; vehicle control 1%) or increasing concentrations (0.01–0.1 mM) of the synthesized hybrids, as well as SAC and caffeic acid (Lead compounds) and 5-fluorouracil (5-FU; the standard drug). After treatment, the cells were fixed with trichloroacetic acid (50% v/v) (MERCK) for a period of one h at 4 °C. The cell proteins were determined by staining with 0.4% (w/v) SRB (Sigma-Aldrich, United States), then they were washed with 1% acetic acid for the removal of unbound SRB and left for air-drying. Protein bound SRB was solubilized in 10 mM Tris-base and the absorbance was measured at 492 nm in a microplate reader (Mindray MR-96A) (33). All of the experiments were performed at least in quintuplicate.

*Antiproliferative activity*

Antiproliferative effect of the most active compounds was also tested through Sulforhodamine B (SRB) assay. Briefly, the cells were seeded to a ﬁnal density of 2500 cells/well in 96-well tissue culture plates and incubated in the same conditions described for viability. The cultures were allowed to grow for 24 h and then were treated with increasing concentrations of the selected hybrids (0.1 – 0.55 mM, ranges depended on the IC_50_ -50% inhibitory concentration- values) or DMSO (vehicle control, 1%), for 0, 2, 4, 6, and 8 days. Culture media was replaced every 48 h. After each incubation time, the cells were fixed, stained, and read as previously described for this technique (32).

*Measurement of Mitochondrial Membrane Potential (ΔΨm)*

Mitochondrial membrane permeability changes were assessed through the fluorescent dye DiOC_6 _(3,3’-dihexyloxacarbocyanine iodide, Thermo Fisher Scientiﬁc, Waltham, MA, USA), and propidium iodide (PI). The cells were seeded to a ﬁnal density of 2.5 x 10^5^ cells/well in 6-well tissue culture plates and were allowed to grow for 24 h. Then, they were treated with hybrids **6e**, **9a**, **9b**, **9c,** and **9e **with its respective IC_50_ (0.18, 0.12, 0.12, 0.11, and 0.12 mM, respectively), being harvested by scrapping at 48 h in the same culture mean, and stained with DiOC_6 _and PI at room temperature for 30 min in darkness. The cells were collected to analyze 10,000 events by ﬂow cytometry with excitation at 488 nm and detection of the emission with the green (530/15 nm) and the red (610/20 nm) ﬁlters. This method allowed us quantifying cells with depolarized mitochondrial membrane (34). 

*Cell cycle analysis*

Cell cycle distribution was analyzed by labelling cells with propidium iodide (PI). Assays were carried out as described by Nicoletti *et al.* (1991). In brief, cells were seeded in 6-well tissue culture plates at a density of 2.5 x 10^5^ cells/well, incubated at 37 °C in a 5% CO_2_ atmosphere. The cultures were allowed to grow for 24 h and then were treated for 48 h with 1% DMSO (vehicle control) or hybrids **6e**, **9a**, **9b**, **9c,** and **9e **with the IC_50_ for each compound (0.18, 0.12, 0.12, 0.11, and 0.12mM, respectively). After the treatment, the cells were collected by scraping and the centrifuged cell pellet was resuspended with phosphate buffered saline (PBS). The cell suspension was fixed in 1.8 mL 70% ethanol at 4°C overnight, afterward, these were centrifuged, washed twice in PBS and resuspended in 300 µL of PBS containing 0.25 mg/mL RNAse (Type I-A, Sigma-Aldrich, Germany) and 0.1 mg/mL PI. Following the incubation in the dark at room temperature for 30 min, the PI ﬂuorescence of 10,000 cells was analyzed using a FACS Canto II ﬂow cytometer and the software BD FACS Diva 6.1.3. (BD Biosciences, San Jose). PI signal was analyzed with excitation at 488 nm, using a Sapphire laser, and fluorescence was detected at 610nm. The cell clumps were excluded with the PI-Area vs PI-Width signals. The cell cycle model was ﬁxed using the software FlowJo 7.6.2 (Ashland, OR, USA), applying the Dean-Jett-Fox model (34, 35). 

*Statistical analysis*

All experiments were performed at least three times. The data are reported as mean ± SE (standard error). Statistical differences between the control group (non-treated) and treated cells were evaluated by one-way ANOVA followed by the Dunnett′s test. Values with *p*  ≤ 0.05 were considered significant. The data were analyzed with GraphPad Prism version 7.04 for Windows (Graph Pad Software, San Diego, California, USA).

**Figures. 3:**
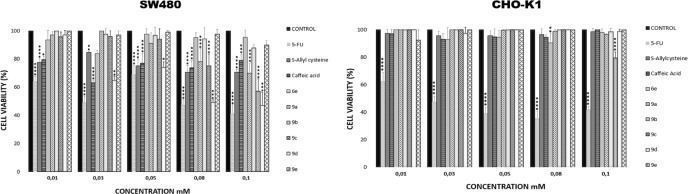
Effect of S-allyl cysteine ester - cafeic acid amide hybrids on cell viability of SW480 and CHO-K1 cells, 48 h post-treatment with different concentrations (0.01-0.1mM). Cell viability was calculated using 100% viability of control. Data are presented as the mean ± SE of three independent experiments (*P < 0.05; **P < 0.01; ***P < 0.001; ****P < 0.0001).

**Figure 4:**
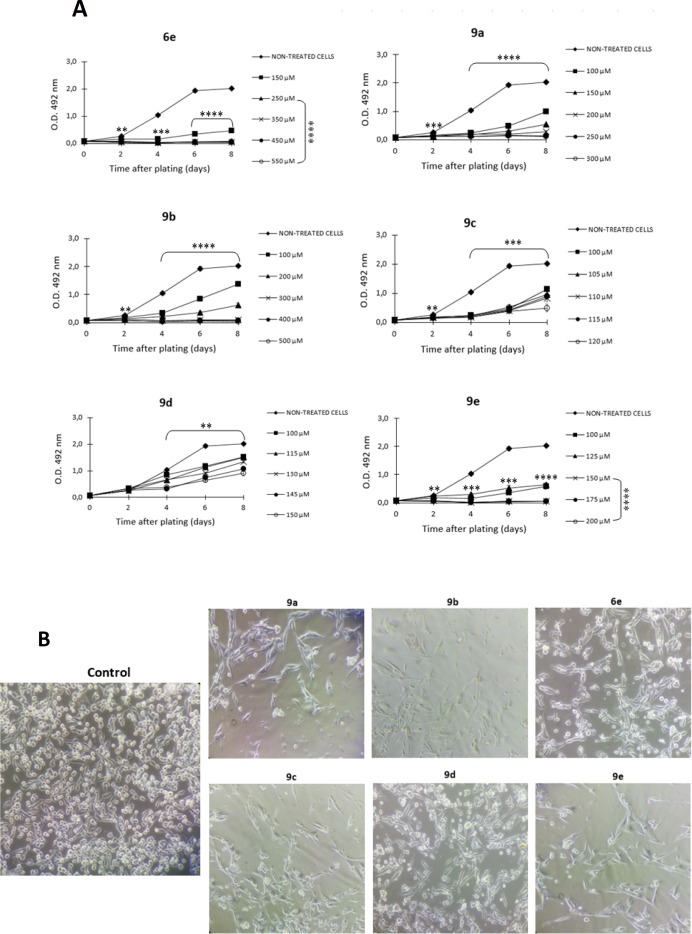
Antiproliferative effect of S-allyl cysteine ester - caffeic acid amide hybrids on SW480 colon cancer cell growth. A) Results obtained with sulforhodamine B assay. B) Representative images of SW480 cells 48 h after treatment with the IC_50_ value (Magnification: 20x). Data are presented as the mean ± SE of at least three independent experiments (**p < 0.01; ***p< 0.001; ****p < 0.0001). Optical Density (O.D.) is directly proportional to cell mass of adherent cells

**Scheme 1:**
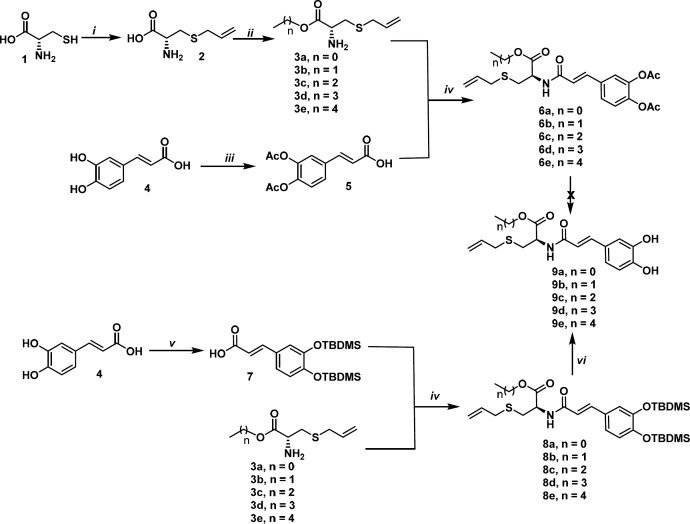
Synthesis of S-allyl cysteine ester-caffeic acid amide hybrids

**Figure 5:**
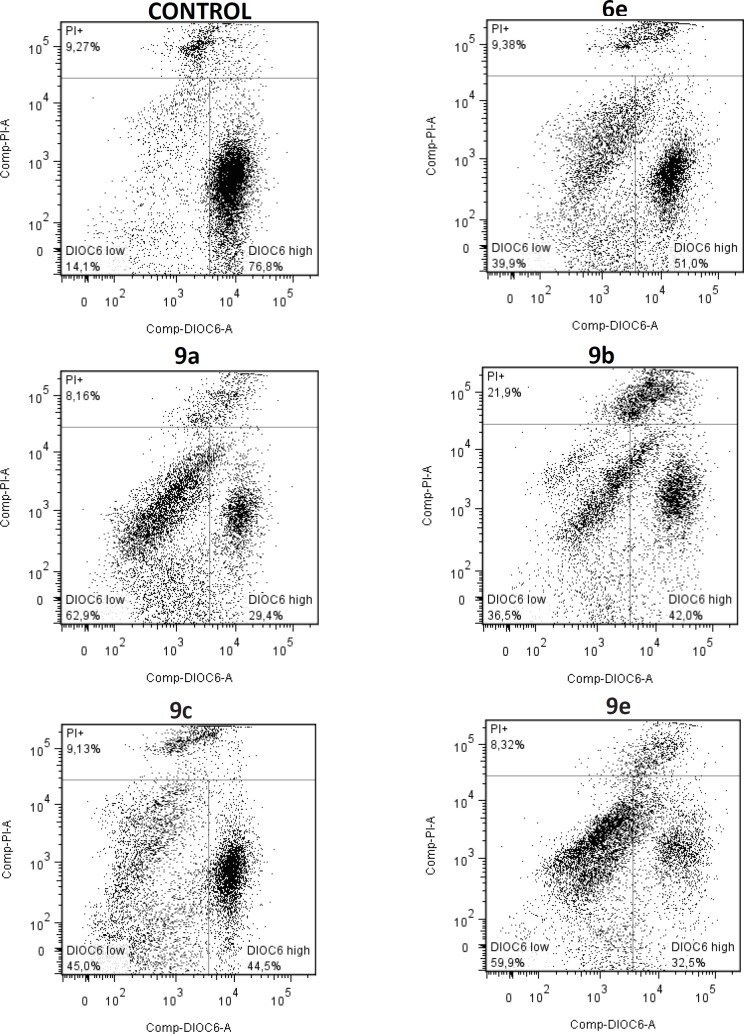
Mitochondrial membrane potential (ΔΨm) in SW480 cells treated with either the most active hybrids or DMSO (1%) as control. DiOC6 high: live cells with high membrane polarization; DiOC6 low: cells in latency that lose membrane polarization; PI +: cells started to lose membrane polarization and in process of death

**Figure 6:**
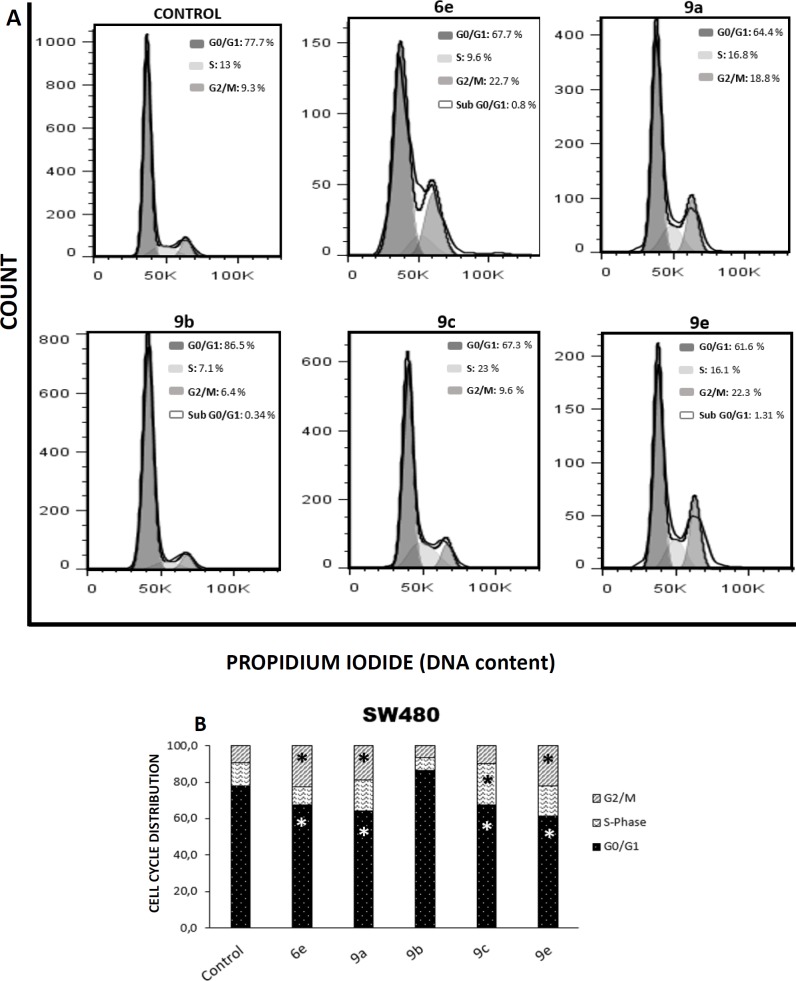
Effect of S-allyl cysteine ester - caffeic acid amide hybrids on cell cycle distribution. (A) Flow cytometry analysis of cell cycle distribution in SW480 cells. (B) The cell cycle distribution in SW480 cells after 48 h of treatment with each hybrid or DMSO 1% (control). *p*-values lower than 0.05 were considered statistically significant (*p< 0.05)

**Table 1:** Cytotoxic effect of S-allyl cysteine ester-caffeic acid amide hybrids on SW480 and CHO K1 cell lines

**Compounds**	**24 h**			**48 h**		
	**IC** **50 ** **(mM)** **CHO-K1 cells**	**IC** **50 ** **(mM)** **SW480 cells**	**SI**	**IC** **50 ** **(mM)** **CHO-K1 cells**	**IC** **50 ** **(mM)** **SW480 cells**	**SI**
**3a**	1.85	>10	<0.19	>10	>10	>1.0
**3c**	1.50	>10	<0.15	1.24	1.28	0.97
**6a**	2.77	0.67	4.2	7.83	>10	<0.78
**6b**	3.84	>10	<0.38	>10	>10	>1.0
**6c**	0.27	1.02	0.26	1.25	>10	<0.13
**6d**	0.21	2.79	0.08	9.97	>10	<1.0
**6e**	**0.26**	**0.68**	**0.40**	1.84****	0.18*	**10.3**
**9a**	**0.47**	**0.24**	**2.0**	0.18**	0.12*	**1.5**
**9b**	**>10**	**0.93**	**>10.75**	>10****	0.12*	**>83.33**
**9c**	**0.12**	**0.10**	**1.2**	>10****	0.11*	**>90.91**
**9d**	**>10**	**0.11**	**>90.91**	>10****	0.15*	**>66.67**
**9e**	**0.21**	**0.20**	**1.03**	>10****	0.12*	**>83.33**
**S-Allylcysteine**	>10	>10	>1.0	>10	0.38	>26.32
**Caffeic acid**	>10	7.06	>1.42	>10	3.47	>2.88
**5-Fluorouracil**	0.091	0.062	1,48	0.048	0.035	1.34

The IC50 values were obtained from dose response curves for each compound. Selectivity index was determined as ratio of IC50 against CHO-K1 to that of IC50 against SW480.

## Results and Discussion

*Chemistry*

The design of the compounds were based on lipophilic modiﬁcations. Allyl cysteine **2** was obtained, in 80% yield, via nucleophilic sustitution between cysteine **1** and allyl bromide (36). Reaction of **1** with the corresponding alcohol in the presence of thionyl chloride (37) afforded, after purification by crystallization or column chromatography, compounds **3a-3e** in 60-90% yields. When these compounds were submitted to peptide type-coupling with 3,4-diacetoxycaffeic acid **5 **(38) using HBTU as amide bond promoter (39), the compounds **6a-6e** were achieved in 30-40% yields. 

On the other hand, the phenolic hydroxyl groups of caffeic acid were protected as TBDMS ethers upon the reaction of reacting caffeic with TBDMSCl and imidazole in a solvent-free reaction assisted by microwaves. In this conditions compound **7** was obtained in 50% yield (40). Then, the compound **7** was coupled with allylcysteine esters **3a-3e** in the same conditions as above to afford amides **8a-8e** in the yields ranging 50-60% (38). Finally, hybrids **9a-9e** were obtained by deprotection from compounds **8** (yields 50-96%) (41). Obtention of hybrids **8** by deacetylation of compounds **6 **(39, 42) was unsuccessful and a complex mixture was observed in all attempts carried out (Scheme 1).

Reagents and conditions: (i) Allyl bromide, NH_4_OH, 80% (ii) SOCl_2_, ROH, -10°C, 60-90%. (iii) Ac_2_O/NaOH, caffeic acid, 80%. (iv) HBTU, Et_3_N, THF, 30-40% (6a-6e), 50-60% (8a-8e). (v) TBDMSCl, Imidazole, caffeic acid, MW, 50% (vi) TBAF, Benzoic acid, dioxane, 50-96% (9a-9e).

The structures of the all compounds have been established by a combined study of IR, ESI-MS, ^1^H-NMR, ^13^C-NMR, Carbon atom types (C, CH, CH_2_, CH_3_), determined by using the DEPT or APT pulse sequence. The signals were assigned using two-dimensional heteronuclear correlations (COSY and HSQC). IR spectra exhibited characteristic absorption peaks corresponding to N-H, C=O, C-O-C and (C=O)-O. ESI-MS spectra showed characteristic [M+H]^+^ peaks corresponding to their molecular weights. The assignments of all the signals to individual H or C-atoms have been performed on the basis of typical δ-values and *J*-constants. The ^1^H-NMR spectra of hybrids **6** and **9** dissolved in CDCl_3_ showed signals of S-C**H**_2_CHN (2.90 and 3.00 ppm), S-C**H**_2_CH=CH_2 _(3.12 ppm), OCH_2_ or OCH_3_ (4.2 or 3.8, respectively), -C**H**-N (4.86-5.03 ppm), S-CH_2_CH=C**H**_2_ (5.10-5.22 ppm), S-CH_2_C**H**=CH_2_ (5.70-5.88), –CO–C**H**=C (6.26 ppm), -CH-N**H**-C=O ( 6.78 ppm), Ar-H (6.80-7,62 Ar-H), and Ar-C**H**=C (7.5 ppm). Additionally, compounds 6 showed signals of acetyl groups ((**C**H_3_-C=O)-O (2.30 and 2.31). ^13^C-NMR spectra of compounds **6** and **8** showed at around 32.60, 35.25, 52.22, 68.13, 116.60, 118.25, 133.60, 143.13, 167.18, 171.80, ppm, corresponding to (S-**C**H_2_CHN), (S-**C**H_2_CH=CH_2_), (CH-N), (OCH_2_ or OCH_3_), ((C=**C**-CO-), (S-CH_2_CH=**C**H_2_), (S-CH_2_**C**H=CH_2_), (Ar-**C**=C), (-NH-**C**=O), and ((CH-**C**=O)-O), respectively. Also, compounds **6** exhibited two signals around 20.76 and 20.79 corresponding to acetyl groups ((**C**H_3_-C=O)-O).

*Biological activity*

*Effect of S-allyl cysteine ester-caffeic acid amide hybrids on SW480 and CHO-K1 cell viability*

In order to assess their effect on the viability, the synthesized S-allyl cysteine ester-caffeic acid amide hybrids were evaluated against SW480 and CHO-K1 cell lines through the sulforhodamine B assay. As shown in Figure 3, the activity was time- and concentration-dependent, with a higher cytotoxic effect on SW480 cells in relation to CHO-K1 cells. Cytotoxicity was reported as 50% inhibitory concentration (IC_50_ values).

All results regarding to the cytotoxic effect are summarized in Table 1. Among the compounds tested, hybrid **6b **was the only one which did not exhibit activity neither at 24 h nor at 48 h after treatment. On the other hand, compounds **6a**, **6c, **and **6d **exhibited good activity against the human colon adenocarcinoma SW480 cells, 24 h after treatment (IC_50_= 0.67, 1.02, and 2.79 mM, respectively); however, the effect decreased after 48 h as evidenced by the rise in the IC_50_ (>10 mM). Moreover, compounds **6e** and **9a-9e **showed the best activity against SW480 cells at 24 h after treatment, with IC_50_ values ranging from 0.10 to 0.93 mM, besides, the activity was maintained through the time since the IC_50_ values decreased 48 h after treatment with IC_50_ values in the range of 0.1-0.18 mM. Additionally, their activity against the normal cell line was significantly lower compared to the reference drug, which gave these compounds great selectivity indexes (**6e**: 10.3; **9a**: 1.5; **9b: **>83.33** 9c: **>90.91** 9d: **>66.67;** 9e**: >83.33) after the 48 h treatment. It is important to highlight that compounds **9b-9e** were much more selective than the lead compounds and the standard drug (5-FU). Compounds **3a** and **3c**, which correspond to allyl esters without hybridization, exhibited higher cytotoxicity against CHO-K1 thus displaying low selectivity indexes. The results regarding the activity of our hybrid compounds are in accordance with those reported by Herrera-R *et al.* (2018), which also found better activity and selectivity indices (SI) with some styrylcoumarin hybrids when tested in SW480 cells (32).  

In the structure-activity relationship (SAR) study, we noticed a synergistic action of the parent subunits when they are linked to form a single structure in the hybrid, as in the case of the compounds **6e** and **9a-9e. **Besides, allylcysteine esters such as **3a** and **3c** show a decreased activity, suggesting that the presence of the acid group is important within the mode of action that could involve the transference of a proton or formation of hydrogen bonds with a receptor (43). In addition, although higher selectivity indexes were achieved when the alkyl chain had two, three, and five carbon atoms (hybrids **9b**, **9c** and **9e**, respectively), there was not a clear relationship between the antitumor activity and the length of the alkyl chain. On the other hand, hybrids with hydroxyl groups (**9a**-**9e**) showed better activity than acetylated derivatives (**6a-6e**), which is in agreement with the reports for several chalcones, coumarins, and caffeic acid esters (44-46), suggesting that this effect could be due to a better molecular recognition ability towards target bioreceptors upon hydrogen bond formation (43), oxidation processes across radical formation, and/or the ability of metal complexation (47). 

*Antiproliferative effect of S-allyl cysteine ester - caffeic acid amide hybrids on SW480 cells*

The most active and selective compounds (**6e**, **9a**–**9e**) were analized through a longer period of time in order to assess if they can display antiproliferative activity. After comparing each treatment with the control, the results indicated that the activity was time- and concentration-dependent. Among the results obtained, hybrids **6e**, **9a**-**9c, **and** 9e** displayed significant antiproliferative activity from day 2 onwards (*p *≤  0.05), even at the lowest concentrations evaluated, while compound **9d** required a longer period of time (4 days) to exert activity against SW480 cells (Figure 4A). Additionally, when observed with optical microscope, the cellular morphology of SW480 cells was severely perturbed, exhibiting changes in size and shape after treatment with S-allyl cysteine ester-caffeic acid amide hybrids, while the control displayed normal and healthy shape. Moreover, there was a clear decreased in number of the cells in comparison with the control, indicating either an increasing progression toward cell death or an arrest in cell cycle (Figure 4B).

*Changes in mitochondrial membrane potential (ΔΨm) induced by S-allyl cysteine ester - caffeic acid amide hybrids*

The changes in Mitochondrial Membrane Potential (ΔΨm) could cause mitochondrial dysfunction, a process that is determinant in the execution of the cell death (48). Therefore, to assess the role of mitochondria in SW480 cells treated with our hybrids, the carbocyanine fluorescent dye DiOC6 was used. This dye accumulates in mitochondria due to its large negative membrane potential and it is released to the cytosol after a membrane depolarization (membrane with reduced ΔΨm), staining intracellular membranes (34, 49). According to the results (Figure 5), all hybrids caused a depolarization in the mitochondrial membrane as regards control, as observed in the decrease of the DiOC6 high population. Besides, in all cases, there was an increase in the field of the cells in latency and with loosing membrane polarization (DiOC6 low). Furthermore, there was not a great population with damaged membrane, which can be appreciated in the field of the cells stained with PI. This behavior was similar for all the evaluated compounds; however, hybrids **9a** and **9e** exhibited the highest activity in this cell line. A possible explanation for this could be related with the ability of hybrids **9a** and **9e** to induce cell death through a cross-talk between the extrinsic and intrinsic pathways of apoptosis via caspase-8 and Bid activation, which leads to mitochondrial membrane permeabilization (50).

*S-allyl cysteine ester - caffeic acid amide hybrids induce cell cycle arrest on SW480 cells*

Numerous studies have suggested that cancer progression involves the loss of checkpoint controls that regulate the passage through the cell cycle (51-54), which is critical in cancer pathogenesis and may affect the effectiveness of chemotherapy (17, 55, 56). Thus, we focused on determine whether hybrids **6e**, **9a**, **9b**, **9c,** and **9e** could have any effect in the regulation of this process. The results obtained show that hybrids **6e**, **9a,** and **9e** caused an arrest in the cell cycle in G_2_/M phase (22.7, 18.8 and 22.3%, respectively). In contrast, the proportion of the cells in G_0_/G_1_ phase significantly decreased with regard to the control (*p* < 0.05). A similar reduction in G_0_/G_1_ phase was observed with compound **9c** (67.3%), along with an important increase in the S-phase of the cell cycle (23%). Additionally, compound **9b** did not have any impact on the cell cycle. These findings complement our above findings, thus suggesting that our hybrids could have not only a cytotoxic effect but also a cytostatic activity (Figure 6).

## Conclusion

Our results demonstrate that some S-allyl cysteine ester-caffeic acid amide hybrids may display antiproliferative activity by inducing mitochondrial membrane depolarization and cell cycle arrest in SW480 cells, being even more active than the lead compounds. Besides, according to the results, the evaluated hybrids exhibited more selectivity than 5-FU, the conventional chemotherapeutic drug. The SAR analysis showed that hydroxyl groups increased the activity, besides, there was not a clear relationship between the antitumor properties and the length of the alkyl chain. All these findings make these hybrid compounds promising candidates for further antitumor studies.
